# Blurred Star Image Processing for Star Sensors under Dynamic Conditions

**DOI:** 10.3390/s120506712

**Published:** 2012-05-22

**Authors:** Weina Zhang, Wei Quan, Lei Guo

**Affiliations:** Science and Technology on Inertial Laboratory, Key Laboratory of Fundamental Science for National Defense-Novel Inertial Instrument & Navigation System Technology, Beijing 100191, China; E-Mails: yuzhanglina@yahoo.com.cn (W.Z.); lguo@buaa.edu.cn (L.G.)

**Keywords:** image denoising, image restoration, star sensor, dynamic conditions

## Abstract

The precision of star point location is significant to identify the star map and to acquire the aircraft attitude for star sensors. Under dynamic conditions, star images are not only corrupted by various noises, but also blurred due to the angular rate of the star sensor. According to different angular rates under dynamic conditions, a novel method is proposed in this article, which includes a denoising method based on adaptive wavelet threshold and a restoration method based on the large angular rate. The adaptive threshold is adopted for denoising the star image when the angular rate is in the dynamic range. Then, the mathematical model of motion blur is deduced so as to restore the blurred star map due to large angular rate. Simulation results validate the effectiveness of the proposed method, which is suitable for blurred star image processing and practical for attitude determination of satellites under dynamic conditions.

## 1. Introduction

As high accuracy and high reliability devices, star sensors play an important role in attitude determination for satellites in celestial navigation system (CNS). The main steps for attitude determination include star point location, star identification and attitude tracking [1]. Based on the captured star images, stars can be located and identified. Whether the attitude determination of satellite is successful or not, the pattern recognition is very important for star images in the field of view (FOV) [2]. It indicates that only the available and recognizable star images can ensure star sensor can give an accurate satellite attitude [3], so it is critical to improve the accuracy of star point location.

In the past many years, some algorithms have been developed to extract star centroids utilizing initial star images. Reference [4] shows a new sub-pixel interpolation technique to process image centroids. Reference [5] gives a method of enhancement of the centroiding algorithm for star tracker measure refinement. An analytical and experimental study for autonomous star sensing, including the star centroid process, is presented in [6]. However, these studies are generally used under static conditions or balanced processes. For many agile maneuver satellites, star sensors work under dynamic conditions as a result of the rotation of the star sensor along with the satellite. Therefore, various noises in star field caused by dynamic factors may affect the quality of imaging. Moreover, due to the large angular rate, the star point moves on the focal plane during the exposure time, which may lead to two changes for the star point: the position shifting on the focal plane and the limited starlight energy dispersing into more pixels. As a result, the SNR (Signal to Noise Ratio) of blurred star images may decrease and the measured star centroid would be inaccurate.

Dynamic conditions stress the need for a very accurate and robust processing method for blurred star images. At present, many investigations tend to concentrate on the exploration and analysis of star location under dynamic condition [3]. For example, reference [7] shows the simulation analysis of dynamic working performance for star trackers. Reference [8] provides an analysis of the star image centroid accuracy of an APS star sensor under rotation conditions. Blind iterative restoration of images with spatially-varying blur is the research topic in reference [9]. However, most of them are limited to the useless locating capability when the angular rate is larger than 2°/s. In [3], the method is effective to estimate attitude, but it has the contrary effect when the angular rate is low.

The main theme of this paper is to overcome the difficulties arising from dynamic imaging blur of star sensors, including denoising and restoration of blurred star images by estimating the angular rate. On the one hand, if the angular rate is in the dynamic range of the star sensor, a proposed adaptive wavelet threshold is used for denoising according to the characteristics of the blurred star image, which can guve the accurate centriod within sub-pixels. On the other hand, if the angular rate is larger than the dynamic range, the restoration algorithm based on the angular rate is used to process the “tail” star image. As will be seen later in this paper, the adaptive method outperforms other denoising methods in terms of Power Signal-to-Noise Ratio (PSNR) and visual qualities, and the large variation of the angular rate has little effect on the star centroid determination based on the restoration method.

This paper is divided into six sections. Following this Introduction, the imaging theory of star sensors is outlined in Section 2, as well as the characteristic of blurred star images under dynamic conditions. Then the method of denoising based on adaptive wavelet threshold is described in detail in Section 3. The restoration method is developed in Section 4 by analyzing the Point Spread Function (PSF) of motion blurred star images, where a Wiener filter with an optimal window is used to overcome the edge error. In Section 5, simulation results are shown to demonstrate the proposed methods. At the end, conclusions are drawn in Section 6.

## 2. Problem Statements

### 2.1. Coordiante Frames

In developing a set of equations to be mechanized by a celestial navigation system and star sensor or in studying the behavior of a given system, it is necessary to introduce several sets of orthogonal coordinates:

- **Inertial coordinate system** (*X_L^-^_Y_L^-^_Z_L_*) has its origin at the center of the Earth and is non-rotating with respect to the fixed stars. Its *x*-axis is in the equatorial plane and the *z*-axis is normal to that plane; and the *y*-axis complements the right-handed system.
- **Star sensor coordinate system** (*X_s^-^_Y_s^-^_Z_s_*) has its origin at the center of mass of the star sensor. Its *x*-axis points along longitudinal axis of the star sensor; the *z*-axis is perpendicular to the longitudinal plane of symmetry and is along the boresight of the star sensor; and the *y*-axis completes a right-handed system.
- **Focal plane coordinate system** (*X_p^-^_Y_p_*) has its origin at the center of the focal plane. Its x-axis points along longitudinal axis of the focal plane; the y-axis is perpendicular to the longitudinal plane.

### 2.2. Imaging Theory of Star Sensor

**Figure 1.**
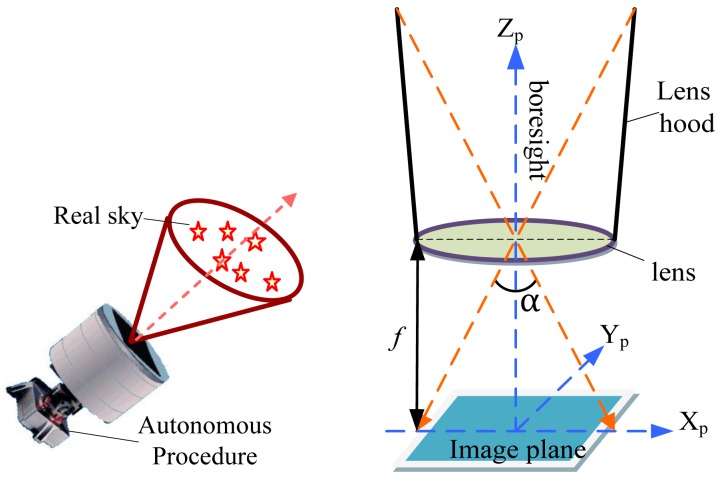
Large FOV star sensor for attitude determination (left) and imaging schematic (right).

Figure 1 illustrates the general large FOV star sensor for attitude determination. After capturing stars in the real sky and imaging by the star sensor, the attitude information is completed by an autonomous procedure (including image pretreatment, star centroiding, star map matching, attitude determination, *etc.*). The schematic of imaging is also shown in Figure 1, where *f* is the focal length of lens and *α* is the angle of FOV.

According to the coordinates of star points in the focal plane coordinate system *X_p^-^_Y_p_*, it is easy to obtain the coordinates matrix ***S*** of star points in the star sensor coordinate system *X_s^-^_Y_s^-^_Z_s_*. Combined with stars coordinates ***G*** in the inertial coordinate system *X_L_-Y_L_-Z_L_*, the attitude ***A*** can be determined as the form of 3 × 3 direct cosine matrix by:

(1) $$A=G^{-1}S$$

### 2.3. Characteristics of Star Image under Dynamic Conditions

Under static conditions, the distribution of star points is generally represented as a two-dimensional Gaussian with a 3 × 3 or 5 × 5 dispersion circle by defocusing technology [10], so that the accuracy of star centroid can be kept within a sub-pixel level. However, under dynamic conditions, the original star image is perturbed and blurred by various additive noises, which mainly include photon response uniform noise, photon shot noise, dark current noise, readout noise, *etc.* [11].

At present, the dynamic range of a large FOV star sensor is about 3–5°/s [8]. Suppose the angular rate is *w*. If *w* ranges by 1–1.5°/s under dynamic condition, the rotation of the star sensor has little effect on the star images. However, a star sensor may lose tracking using the technique under static conditions due to the various noises caused by the dynamic conditions. On the other hand, if *w* is larger than the dynamic range, the star point constantly shifts in the focal plane and appears to trail badly during exposure time, which may affect the star centroid accuracy and even result in the failure of attitude determination.

Based on the foregoing discussions, denoising and deblurring are two crucial parts for the pretreatment of blurred star images. Performance parameters of the star sensor used in this paper are shown in Table 1. The CMOS image sensor chip is the STAR 1000 from the Cypress Semiconductor Co. [12].

## 3. Blurred Star Image Denoising

### 3.1. Denoising Modeling Based on Wavelet Transform

Supposing the size of the clear image *f(i, j)* is *N* × *N*, a common model of the corrupted image *g(i, j)* is mathematically defined as:

(2) g(i,j)=f(i,j)+n(i,j)

where 0 ≤ *i, j* ≤ *N*-1, and *n(i, j)* is additive random noise and independent of *f(i, j)*. The goal is to remove *n(i, j)* and estimate *f(i, j)* which minimizes the Mean Squared Error (MSE) [13].

In general, the important information of *f(i, j)* is mostly distributed as a smooth signal at low frequency, while *n(i, j)* is distributed at high frequency. Based on this, a two-dimensional (2-D) discrete wavelet transform (DWT) can be implemented to transform *g(i, j)* into the wavelet domain. Then, wavelet coefficients denoting different scales and orientations can be obtained with the use of the Mallet algorithm [14].

**Figure 2.**
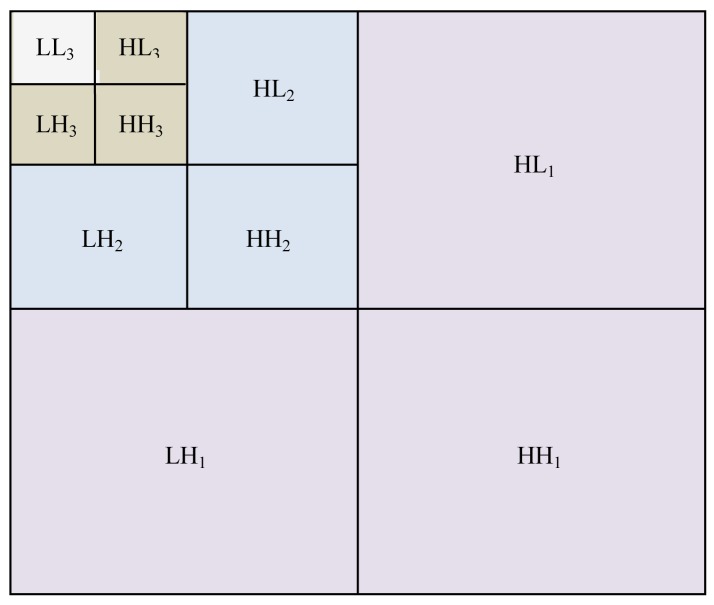
Subbands of the 2-D orthogonal wavelet transform.

Figure 2 shows the subbands of the orthogonal DWT of three levels. *LL_3_* is an approximation subband (or the resolution residual) which contains the low frequency portion of *g(i, j)*.The subbands *HL_k_*, *LH_k_*, *HH_k_* (*k* = 1,2,3) respectively denote the details of vertical, horizontal and diagonal orientations, where *k* is the scale and size of a subband at scale *k* is *N*/2*^i^* × *N*/2*^i^*. There is no space here to go into detail on this method, and for this level of detail the reader can refer to [13,15] for more information. It is important to point out the small coefficients mostly due to noise and large coefficients due to important signals. Accordingly, denoising can be accomplished by thresholding these coefficients.

### 3.2. Threshold Selection

Thresholding is simple because it operates on one wavelet coefficient at a time. The method of using an adaptive threshold to implement denoising described by Lakhwinder Kaur *et al.* [16] appears more suitable, in which threshold choice is:

(3) $$T_{N}=\frac{\beta \sigma^{2}}{\sigma_{y}}$$

where *σ_y_* is the standard deviation of each subband, and *β* is the scale parameter for each scale computed by:

(4) $$\beta =\sqrt{\log\frac{L_{k}}{J}}$$

where *J* is the largest scale, and *L_k_* is the length of subband at the scale of *k* (*k* = 1,2…*J*). The noise variance σ^2^ is obtained by:

(5) $$\sigma^{2}=\text{Median}(|HH_{1}|)/0.6745$$

Studies in [17] indicate that the square error relating to *HH_1_* of *g(i, j)* almost equals the noise variance *σ*^2^. On the other hand, the larger the decomposition level is, the smaller the weight of noise in the coefficient variance will be. For this reason, it is more convenient to complete denoising for the star images than general images because the contrast between star signal on a black background and noise is more distinct, even when the star point is blurred under dynamic condition. We pay attention to *T_N_* in scale *J*, where has the approximation of star points. This proposed method processes each coefficient in scale *J* using a different threshold. It can be executed mainly by the following steps:

1. Apply an M × M local window to compute σ^2^_lJ_, which denotes the coefficients variance of window l in scale J. M is determined by the square root of the number of pixels occupied by the star point, and generally is not more than seven.
2. Compute noise variance σ^2^ according to Equation (5).
3. Obtain the threshold by:
  (6) $$Th_{i}=\beta \frac{\sigma^{2}}{\sigma_{lJ}^{2}}$$
  where *Th_l_* is the threshold in window *l* of scale *J*.

### 3.3. Star Image Denoising

Based on the foregoing analysis, the proposed method for star image denoising is summarized as follows:

1. Execute decomposition of the initial blurred star image using a wavelet transform at level K.
2. Compute the noise variance σ^2^ according to Equation (5).
3. Compute the scale parameter β of level K using Equation (4).
4. Use a 4 × 4 square window in LLK to obtain Thl by Equation (6).
5. Process coefficients in scale K using the following threshold function:
  (7) $$\hat{x}=\{\begin{array}{cc} \mathrm{sgn}(x)(|x|-Th_{l}), & |x|\geq Th_{l}\\ 0, & |x|\leq Th_{l} \end{array}$$
  which keeps the coefficients information if it is larger than threshold; otherwise, it is set to zero.
6. Invert the multiscale decomposition to reconstruct the denoised star image.

## 4. Blurred Star Image Restoration

The current angular rate w of satellite can be obtained by the attitude update. As mentioned in Section 2, if *w* is larger than the dynamic range, the attitude cannot be correctly computed because of the “trailed” image. This section mainly focuses on the restoration of motion-blurred star image as a result of large *w*.

### 4.1. Mathematical Model of Blurred Star Image

Due to the motion of the star sensor during exposure time, what a star sensor captures is a motion-blurred image *g(x, y)*. Suppose, a clear image *f(x, y)* moves on the focal plane, its displacement components of direction *x* and *y* can be respectively termed as *x(t)* and *y(t)*, where *t* is the movement time during exposure time *T*. Then, the expression of *g(x, y)* can be obtained from Equation (8), and the expression of PSF in frequency domain can be obtained from Equation (9), which is similar as a previous reference [18]:

(8) $$g(x,y)=\int_{0}^{T}f(x-x(t),y-y(t))dt$$

(9) $$H(u,v)=\int_{0}^{T}e^{-j2\pi [ux(t)+vy(t)]}dt$$

**Figure 3.**
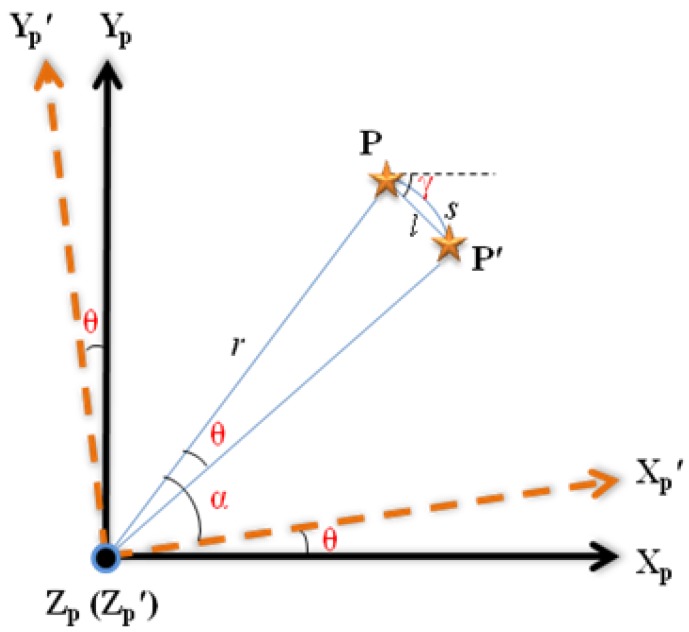
Procedure of star image motion-blurred.

If the satellite rotates clockwise about the boresight *Z_p_* with an angular rate *w_z_* during exposure time *T*, it seems that the star point displaces anticlockwise on focal plane, which results in the trail effect. The motion-blurred procedure can be illustrated in Figure 3.

where *X_p^-^_Y_p_* is the coordinate system of focal plane, *X_p_*′*-Y_p_*′ is the corresponding coordinate of *X_p^-^_Y_p_* after rotation, and *θ* = *w_z_t*, where *t* is the rotation time in *T*. The star point *P* shifts to *P*′ along the circular arc *s* with radius *r* in the focal plane. It is reasonable to assume that the rotation axis and the motion parameters are constants, and *l* is approximate to *s*. As a result *l* can be expressed as:

(10) $$l=rw_{z}t$$

where $r=\sqrt{{x_{p}}^{2}+{y_{p}}^{2}}$, and (*x_p_*, *y_p_*) is the coordinates of *P* in coordinate system *X_p^-^_Y_p_*.

Suppose *P* moves along track *l* with angle *γ* to the horizontal axis *X_p_* by velocity *v*, where *v* = *w_z_r* and *γ* can be obtained by *θ*, *x_p_* and *y_p_*. Combined with Equation (8), the PSF of motion blurred star image can be obtained by:

(11) $$H(u,v)=\frac{T\sin(\pi \text{ul}\cos\gamma )}{\pi \text{ul}\cos\gamma }e^{-j\pi \text{lu}\cos\gamma }+\frac{T\sin(\pi \text{vl}\sin\gamma )}{\pi \text{vl}\sin\gamma }e^{-j\pi \text{lv}\sin\gamma }$$

Then, the expression of *H(u,v)* in the time domain is:

(12) $$h(x,y)=\{\begin{array}{ll} 1/l & 0\leq x\leq l\cos\gamma ,y=x\tan\gamma \\ 0 & \text{else} \end{array}$$

### 4.2. Blurred Star Image Restoration

To accomplish restoration of the original image, the traditional method is to employ Wiener Filtering in the frequency domain [15]. The Wiener filter is intended to be an optimal filter in the sense that it delivers the best estimate of the object in a least-squares sense for additive Gaussian noise. However, the noise *n(x, y)* is typically unknown in practice and the classical Wiener filter is problematic [19]. Therefore, we use the modified Wiener filter which is given by:

(13) $$\hat{F}(u,v)=\frac{H^{*}(u,v)}{H(u,v)H^{*}(u,v)+a}G(u,v)$$

where H*(*u, v*) denotes the complex conjugate of *H*(*u*, *v*) and *a* can be considered as an adjustable empirical parameter chosen to balance sharpness against noise.

In order to overcome the edge error, a major factor affects the quality in Wiener filter restoration, the optimal window method is used for star image [20]. Then the steps of restoration based on Wiener filtering are detailed as follows:

1. Introduce *h(x,y)* according to analysis in Section 4.1.
2. Apply the optimal window *w*(*x, y*) as a weight factor to *g*(*x, y*), then execute the Discrete Fourier Transform (DFT) of *g*(*x, y*) and *h*(*x, y*).
3. Use the Wiener filter for deconvolution filtering in the frequency domain, and obtain the estimate of *F*(*u, v*) by Equation (13).
4. Compute the Inverse DFT (IDFT) of *fˆ*(*x*,*y*) to generate *f*(*x, y*) by:
  (14) $$f(x,y)=\int_{-\infty }^{+\infty }\int_{-\infty }^{+\infty }F(u,v)e^{j2\pi (ux+vy)}d_{u}d_{v}$$

## 5. Results and Analysis

In order to verify the proposed method when a star sensor works under dynamic conditions, simulations and experiments are implemented to accomplish denoising and restoration according to blurred star images caused by different *w*. Comparison of PSNR and the star centroid are also analyzed to estimate the effect of algorithm in this section.

### 5.1. Denoising of Blurred Star Image

**Figure 4.**
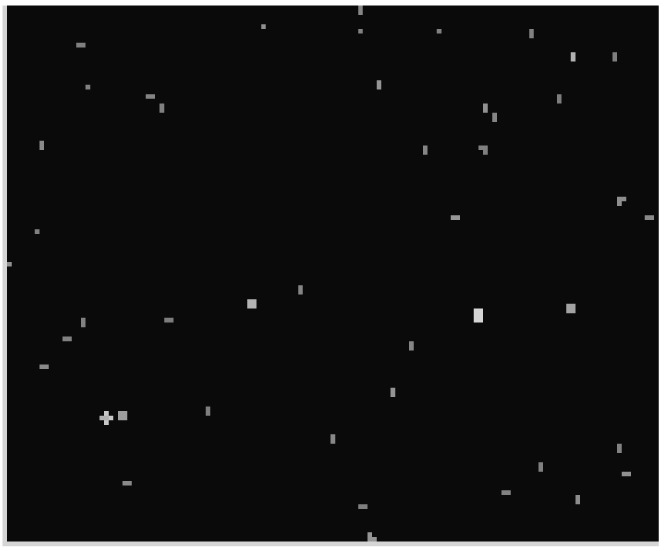
The original star image by simulation.

Based on the performance of the star sensor shown in Table 1, the SkyMap star map simulation software is used to generate the original star image, as shown in Figure 4. The boresight direction is set as (150°, 15°) and the 14,581 stars brighter than 6.95 m are selected in Tycho2n star catalog.

The experiments are conducted on several blurred star images at different noise levels *σ* = 70, 80, 90 and different angular rates *w* under the dynamic range. For the wavelet transform, four levels of decomposition are used, and the wavelet employed is sym8 (from the MATLAB wavelet toolbox).

**Figure 5.**
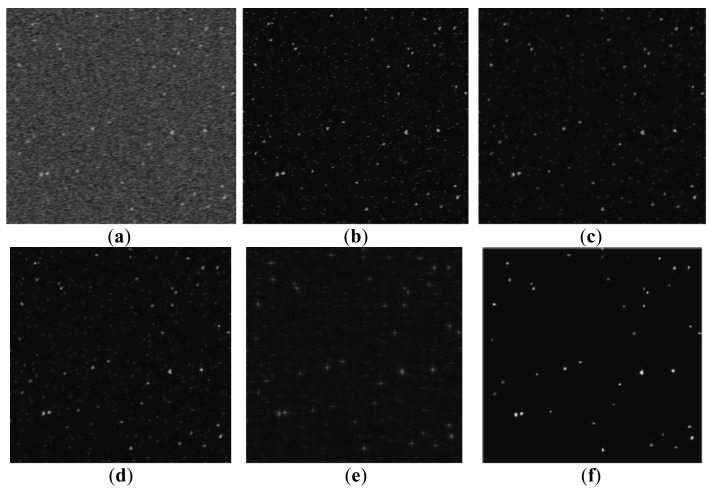
Comparison of noisy image and resulting images. (**a**) Noisy image at *σ* = 90, *w* = 0.6°/s, (**b**) Resulting image with fixed threshold, (**c**) Resulting image with BayesShrink, (**d**) Resulting image with SureShrink, (**e**) Resulting image with Lowpass filter, (**f**) Resulting image with AdaptThr.

To assess the performance of the denoising method proposed in this paper, it is compared with several common denoising techniques like BayesShrink [13], SureShrink [21] and Lowpass filter. The fixed threshold *Th* is used first to segment the background and the star object. Based on *Th*, different denoising methods are employed to estimate the original clear star image. Figure 5 shows the noisy image and resulting images at *σ* = 90 and *w* = 0.6°/s. We can see that the image processed by the proposed method outperforms the others in terms of visual quality. Then, the PSNRs from various methods are compared in Table 2, and the data are collected from an average of four runs. The *AdaptThr* method, namely, is the proposed adaptive thresholding method.

Results in Table 2 show that the lower *w* is, the better *AdaptThr* performs than other methods, especially when *σ* is large. *AdaptThr* approximately has the same poor effect of denoising along with the increase of *w*. Actually, in dynamic condition with high *w*, star image is not only perturbed by various noises, but also is blurred by the motion of star sensor.

**Figure 6.**
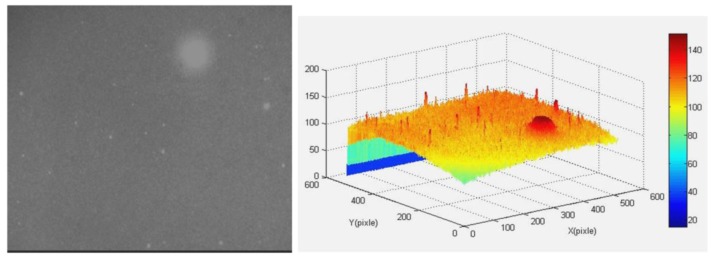
The original real star image and its gray distribution.

**Figure 7.**
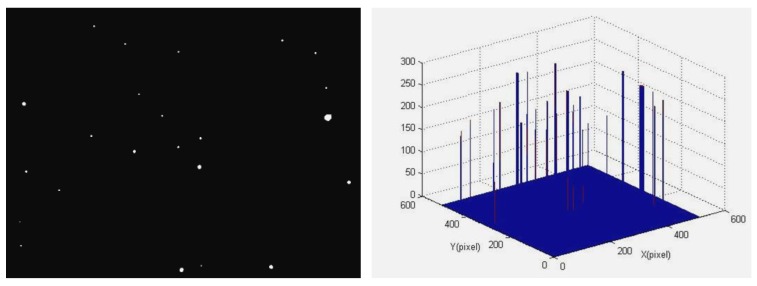
Star image and its gray distribution after discarding singularity spot.

This also means that by only using the proposed denoising method under dynamic conditions with high *w*, one cannot obtain the star centroid accurately, and one also needs to restore the motion-blurred image. In order to further verify the proposed denoising algorithm, a real star image is adopted in this section. Figure 6 shows the original star image obtained by a star sensor and its gray distribution, from which we can see that the background value in the star image is large. What's more, there is a big ‘singularity spot’ which is larger and lighter than other star points. After discarding the singularity spot, a clear star image can be obtained as shown in Figure 7. It can be seen that the dim star object is extracted perfectly from the background noise. This confirms the notable effect of the proposed method which can adapt to the complex dynamic conditions.

### 5.2. Restoration of Blurred Star Image

**Figure 8.**
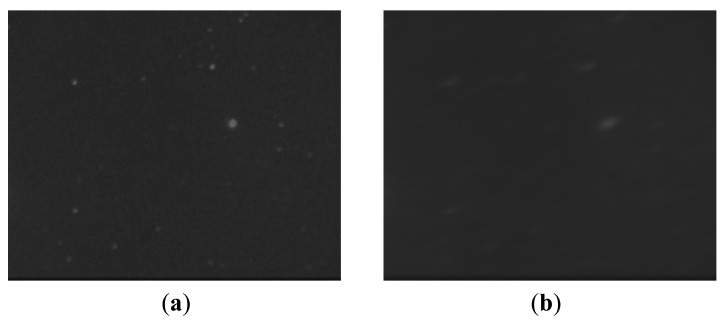
Star images. (**a**) Real star image (**b**) Blurred star image.

Table 2 shows that if *w* is larger than the dynamic range, star image needs to be restored by deblurring rather than by denoising directly. Figure 8(a) is a real star image slected from the original images obtained by the CMOS star sensor in this paper. Based on the supposition *w* = 10°*/s*, the blurred star image can be generated according to the degradation model, as shown in Figure 8(b).

**Figure 9.**
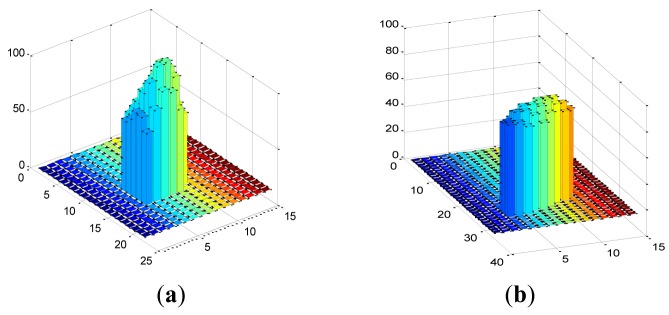
Gray distribution of the same star point. (**a**) Gray distribution of original star point; (**b**) Gray distribution of blurred star point.

Gray distributions of the same star point in two different images of Figure 8 are respectively shown in Figure 9, which show that due to the motion blur, the star point smears out intensely, as well as the gray value decreases.

In this section, we implement star centroiding [2] to assess the performance of the proposed deblurring method. The comparison results are shown in Table 3, where the angular rate is 10°/s.

**Figure 10.**
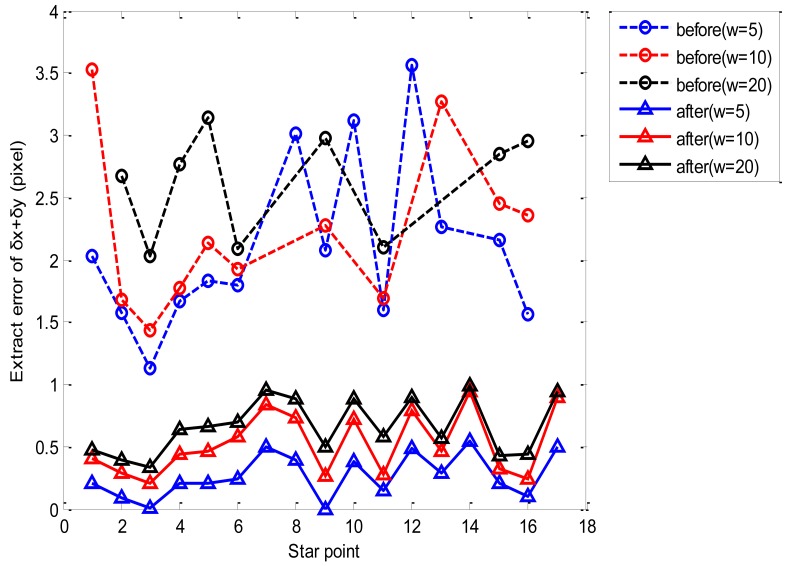
Extraction error of star centroid with different w.

Table 3 shows that the extraction errors of *δx* and *δy* are mostly larger than a pixel for each star centroid without deblurring. This is because the SNR of star image decreases as a result of the star points smearing significantly. Moreover, six star points fail to be extracted due to the low gray value of dim blurred star points (as shown in Figure 9(b)), which may affect the star recognition and attitude determination. However, after restoration in advance, the star centroid can be obtained accurately for in that the extraction errors of *δx* and *δy* are within subpixel range, as well as the dim star points with low gray value are extracted. As can be seen in Figure 10, the extraction error of *δx* + *δy* error is larger than three pixels. With the restoration method, all lost star points can be extracted as well as the extraction error of *δx* + *δy* is restricted within one pixel. This is because the proposed deblurring method can keep the accuracy of *δx* and *δy* within subpixel levels, even when *w* is larger than the dynamic range, and the variation of angular rate *w* has little effect on the star centroid.

## 6. Conclusions

This article researches how to process blurred star images according to different angular rates of star sensors under dynamic conditions. A new denoising method based on adaptive wavelet threshold is proposed, as well as a restoration method according to large angular rate out of the dynamic range. Experiments on different types of star images have been conducted with the proposed algorithm. The PSNRs of images with different types of angular velocity show the proposed denoising method, in comparison with the normal denoising methods, has good performance, namely, better than PSNRs of other methods under the same conditions when the angular velocity is in the dynamic range, and also in terms of visual quality. Star centroiding against blurred star images have been analyzed to assess the effectiveness of restoration. It is confirmed that the restoration maintains the extraction error within subpixel levels, and the variation of angular velocity has little effect on the accuracy of star centroid, which shows that the proposed method is both effective and feasible. Experimental results show that the processing method according to angular velocity in before/after using the restoration method with different angular velocity are analyzed, and star points which can be extracted in each method are also shown. Without restoration, the larger the angular velocity is, the more star points cannot be extracted while the extraction under dynamic conditions reported in this paper could keep star sensors stable within a certain range and meet the requirements of attitude determination, which needs uninterrupted output data and attitude accuracy of arcsecond level.

## Figures and Tables

**Table 1.** Performance parameters of CMOS star sensor [12].

Performance parameters	Value	Unit
*Sensitive area format*	1,024 × 1,024	Pixels
*FOV*	20 × 20	°
*Pixel size*	15 × 15	Mm
*Focal length*	54.646	Mm
*Exposure time*	200	Ms
*Radius of Gaussian PSF*	1	*σ_PSF_*
*Dynamic range*	2	°/s
*Aperture*	40	Mm
*SNR after defocused*	5.5	–

**Table 2.** Comparison of PSNR for each method with various σ and w.

**Method**		**Noise image**	**Fixed threshold (Th = 128)**	**BayesShrink**	**SureShrink**	**Lowpass filter**	**AdaptThr**

*w* = 0.1	*σ* =70	22.15	25.27	28.56	28.89	30.15	35.20
	*σ* =80	17.57	24.78	27.78	27.15	28.53	34.03
	*σ* =90	15.20	24.42	27.43	26.45	27.85	32.56

*w* = 1	*σ* =70	17.52	24.52	26.15	26.53	26.34	32.79
	*σ* =80	16.13	23.68	25.42	24.78	25.89	32.21
	*σ* =90	14.00	23.06	24.77	24.02	25.09	30.04

*w* = 5	*σ* =70	15.73	20.79	20.46	20.71	21.16	22.23
	*σ* =80	14.08	20.23	18.58	19.33	19.54	20.56
	*σ* =90	12.42	17.01	17.17	17.07	18.12	18.16

**Table 3.** Comparison of star centroiding against blurred star image.

**Index of star point**	**Ideal coordinates (pixel)**	**Coordinates of blurred star image (pixel)**	**Coordinates of restored star image (pixel)**
1	(1.4757, 17.4887)	(2.5754, 15.0621)	(1.6321, 17.7413)
2	(61.1800, 247.9956)	(60.3000, 247.2000)	(61.0166, 247.6088)
3	(66.3157, 72.2644)	(65.5620, 71.5855)	(66.5137, 72.4756)
4	(67.0611, 199.5018)	(66.2736, 198.5205)	(67.1176, 199.8830)
5	(106.2782, 235.2798)	(105.0847, 234.3384)	(106.5062,235.5103)
6	(134.6371, 68.7471)	(133.4000, 68.0637)	(134.9628, 69.0000)
7	(150.0000, 218.0000)	Fail	(150.4107, 218.5203)
8	(200.5139, 20.3319)	Fail	(200.8509, 20.9140)
9	(203.0953, 56.8732)	(201.6690, 56.0245)	(203.2726, 56.9546)
10	(201.2458, 251.7475)	Fail	(200.8412,251.3327)
11	(223.0692, 113.0331)	(222.3383, 112.0692)	(223.1885,113.1873)
12	(231.4061, 10.9092)	Fail	(231.8121, 11.4121)
13	(236.5062, 5.5021)	(235.1504, 7.4118)	(236.2813, 5.7401)
14	(244.0000, 58.0000)	Fail	(244.5132, 58.6318)
15	(269.2561, 139.0000)	(268.2537, 137.5502)	(269.3864,139.1944)
16	(271.5072, 114.7826)	(270.5577, 113.3748)	(271.6662,114.8652)
17	(299.0000, 145.0000)	Fail	(299.5125, 145.4218)

## References

[1] Zhang S.D., Zhang Z.J., Sun H.H., Wang Y.J. (2010). High accuracy star image locating and imaging calibration for star sensor technology. Proc. SPIE.

[2] Liebe C.C. (2002). Accuracy performance of star trackers—A tutorial. IEEE Trans. Aero. Elec. Sys..

[3] Wu X.J., Wang X.L. (2011). Multiple blur of star image and the restoration under dynamic conditions. Acta Astronaut..

[4] Brendan M.Q., Valery T., Henok M., Richard H. (2007). Determining star-image location: A new sub-pixel interpolation technique to process image centroids. Comput. Phys. Commun..

[5] Rufino G., Accardo D. (2003). Enhancement of the centroiding algorithm for star tracker measure refinement. Acta Astronaut..

[6] Gwanghyeok J. (2001). Autonomous Star Sensing, Pattern Identification and Attitude Determination for Spacecraft: An Analytical and Experimental Study.

[7] Shen J., Zhang G.J., Wei X.G. (2010). Simulation analysis of dynamic working performance for star trackers. J. Opt. Soc. Am..

[8] Li X., Zhao H. (2009). Analysis of star image centroid accuracy of an APS star sensor in rotaton. Aerospace Control Appl..

[9] Bardsley J., Jefferies S., Nagy J., Plemmons R. (2006). Blind iterative restoration of images with spatially-varying blur. Optics Express.

[10] Hancock B.R., Stirbl R.C., Cunningham T.J., Pain B., Wrigley C.J., Ringold P.G. (2001). CMOS active pixel sensor specific performance effects on star tracker/image position accuracy. Proc. SPIE.

[11] Pasetti A., Habine S., Creasey R. Dynamical Binning for High Angular Rate Star Tracking.

[12] (2007). Star1000 1M Pixel Radiation Hard CMOS Image Sensor.

[13] Chang S.G., Yu B., Vetterli M. (2000). Adaptive wavelet thresholding for image denoising and compression. IEEE Trans. Image Process..

[14] Mallat S. (1989). A theory for multiresolution signal decomposition: The wavelet representation. IEEE Trans. Patt. Anal. Machine Intell..

[15] Gonzalez R.C., Woods R.E. (2002). Digital Image Processing.

[16] Kaur L., Gupta S., Chauhan R.C. Image Denoising Using Wavelet Thresholding.

[17] Mihcak K.M., Kozintsev L., Ramchandran K. (1999). Low-complexity image denoising based on statistical modeling of wavelet coefficients. IEEE Sign. Process. Lett..

[18] Quan W., Zhang W. Restoration of Motion-Blurred Star Image Based on Wiener Filter.

[19] Costello T.P., Mikhael W.B. (2003). Efficient restoration of space-variant blurs from physical optics by sectioning with modified Wiener filtering. Digital Signal Process..

[20] Tan K.C., Lim H., Tan B.T.G. (1991). Windowing techniques for image restoration. Graph. Mod. Image Process..

[21] Donoho D.L., Johnstone I.M. (1995). Adapting to unknown smoothness via wavelet shrinkage. J. Am Stat. Assoc..

